# Supplementary material for: A gut-activated NHR-86–CYP pathway mediates the neuroprotective effects of Enterococcus faecium probiotics in a nematode model of amyotrophic lateral sclerosis
Source: PLoS Biol. 2026 Jan 30;24(1):e3003627. doi: 10.1371/journal.pbio.3003627 (PMC12872002; doi:10.1371/journal.pbio.3003627)
Supplement: S1 Fig — Representative images show animals lacking (A) zero, (B) one, (C) two, and (D) more than 2 motor neurons posterior to the vulva. Animals missing at least two neurons were scored as defective. Missing neurons are marked with red circles. (PDF) [file pbio.3003627.s001.pdf]

S1 Fig

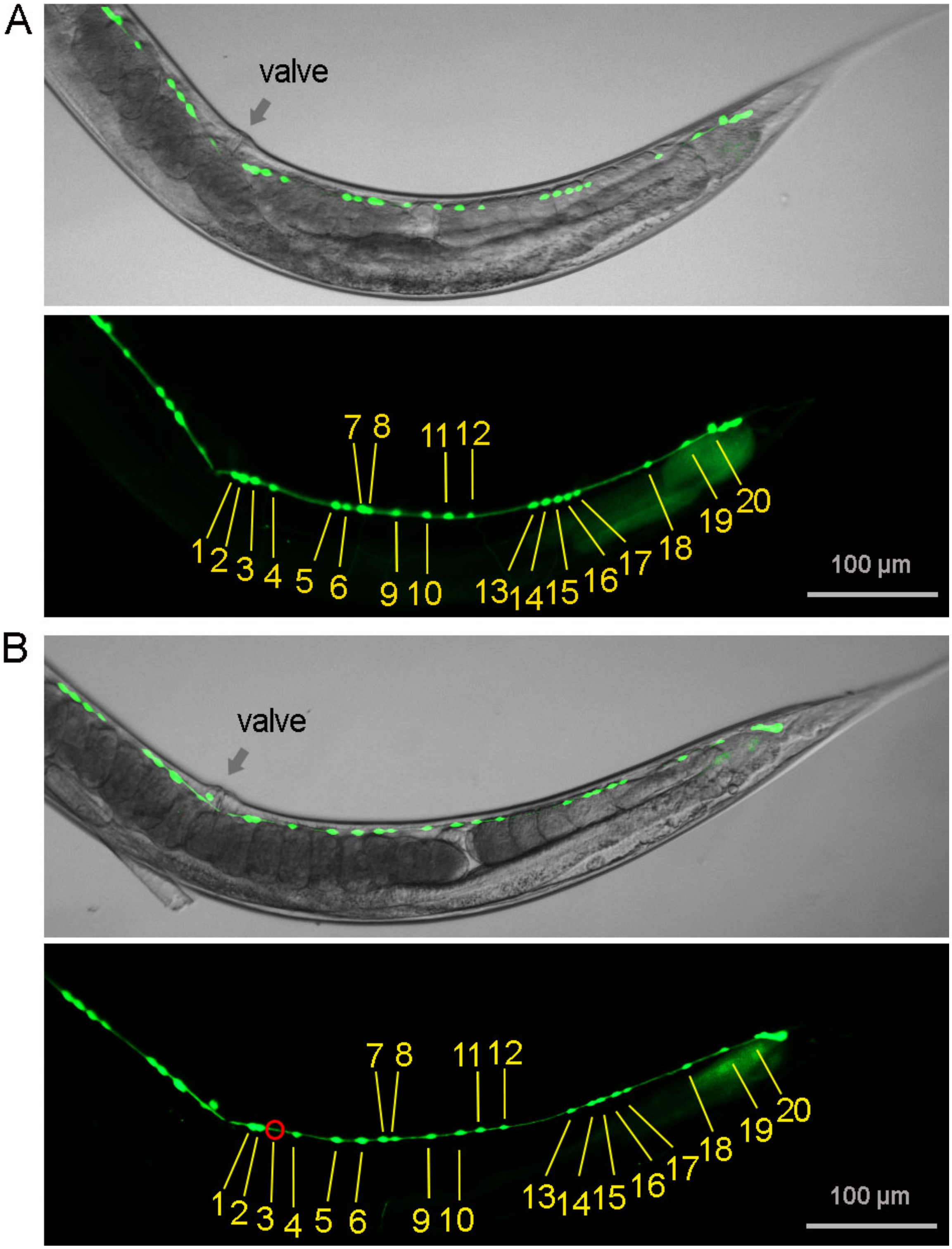
A
valve
7 8
11 12
1 2 3 4 5 6 9 10 13 14 15 16 17 18 19 20
100 µm
B
valve
7 8
11 12
1 2 3 4 5 6 9 10 13 14 15 16 17 18 19 20
100 µm

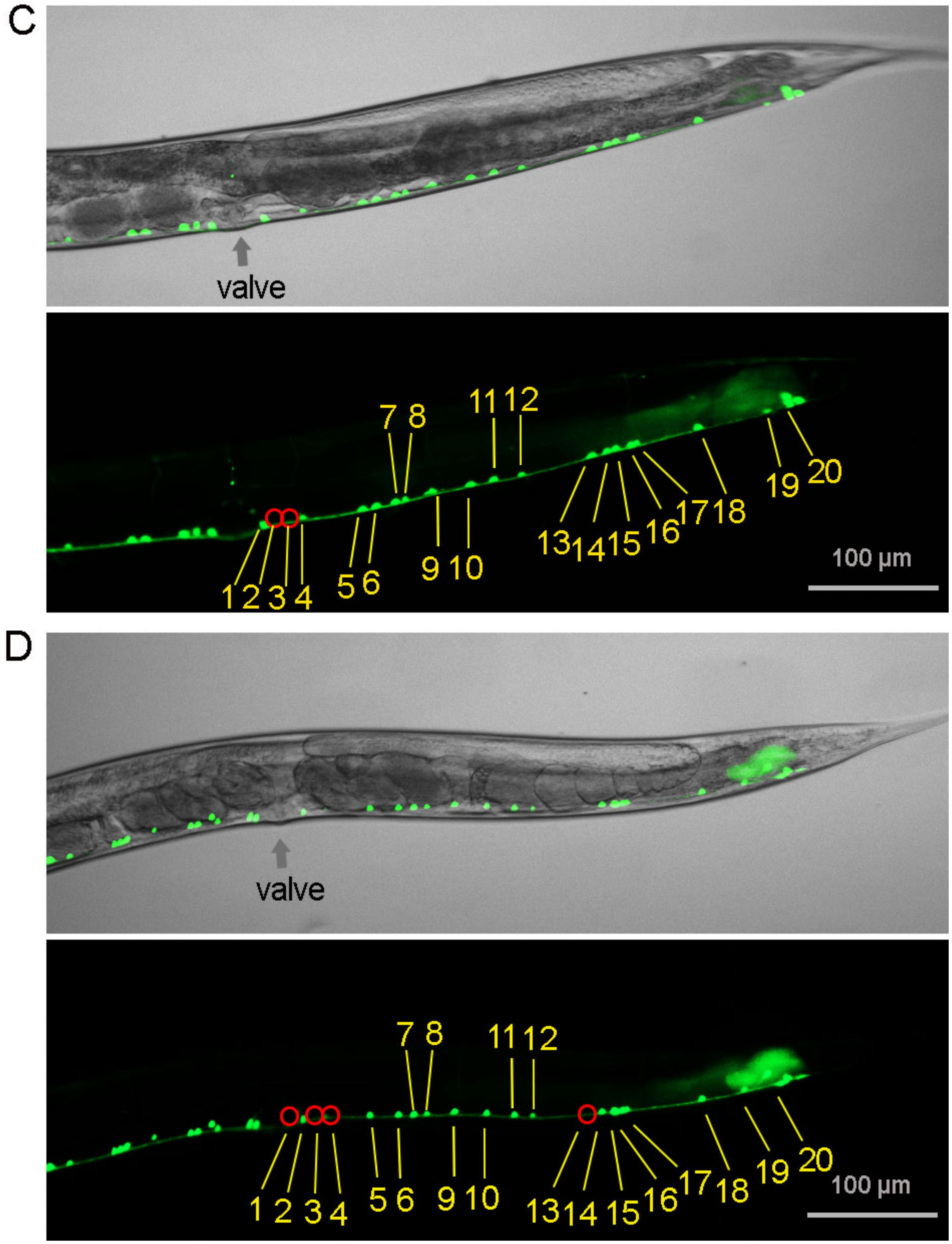

Motor neuron degeneration under paraquat-induced oxidative stress. Representative images show animals lacking (A) zero, (B) one, (C) two, and (D) more than 2 motor neurons posterior to the vulva. Animals missing at least two neurons were scored as defective. Missing neurons are marked with red circles.
