# Supplementary material for: A gut-activated NHR-86–CYP pathway mediates the neuroprotective effects of Enterococcus faecium probiotics in a nematode model of amyotrophic lateral sclerosis
Source: PLoS Biol. 2026 Jan 30;24(1):e3003627. doi: 10.1371/journal.pbio.3003627 (PMC12872002; doi:10.1371/journal.pbio.3003627)
Supplement: S2 Fig — Motor neuron degeneration in sod-1 WTM and in sod-1 A4VM animals was assessed under paraquat-induced oxidative stress, with or without E. faecium pretreatment. Animals missing at least two neurons were scored as defective. A two-tailed Student t test was performed to compare data between different treatments within the same group. (PDF) [file pbio.3003627.s002.pdf]

# S2 Fig

| Animal | Treatment | N | % animals with defective motor neurons | P |
|---|---|---|---|---|
| *sod-1* $WT^M$ | *Ec*-paraquat | 160 | 7.50 | |
| *sod-1* $WT^M$ | *Ef*-paraquat | 166 | 5.42 | 0.1223 |
| *sod-1* $A4V^M$ | *Ec*-paraquat | 127 | 48.03 | |
| *sod-1* $A4V^M$ | *Ef*-paraquat | 126 | 17.46 | 0.0005 |

**Motor neuron degeneration with or without *E. faecium* pretreatment.** Motor neuron degeneration in *sod-1* $WT^M$ and in *sod-1* $A4V^M$ animals was assessed under paraquat-induced oxidative stress, with or without *E. faecium* pretreatment. Animals missing at least two neurons were scored as defective. A two-tailed Student's t-test was performed to compare data between different treatments within the same group. The data underlying this Figure can be found in S1 Data.
