# Supplementary material for: A gut-activated NHR-86–CYP pathway mediates the neuroprotective effects of Enterococcus faecium probiotics in a nematode model of amyotrophic lateral sclerosis
Source: PLoS Biol. 2026 Jan 30;24(1):e3003627. doi: 10.1371/journal.pbio.3003627 (PMC12872002; doi:10.1371/journal.pbio.3003627)
Supplement: S3 Fig — (A) sod-1 A4VM animals were pretreated for 24 hours with live, UV-killed, or heat-killed E. faecium before exposure to paraquat-induced oxidative stress. Cholinergic motor neuron integrity was assessed. Data are represented as mean ± SD from three independent experiments. Statistical significance was determined using one-way ANOVA with Tukey’s multiple comparison test (***p < 0.001). (B) Summary of motor neuron defects from (A) across three independent experiments. (PDF) [file pbio.3003627.s003.pdf]

S3 Fig

A

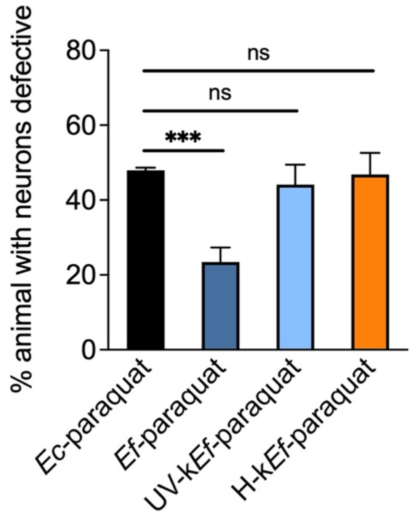

B

Animal	Treatment	N	% animals with defective motor neurons	P
sod-1 A4V^M	*Ec*-paraquat	123	45.87	
sod-1 A4V^M	*Ef*-paraquat	138	19.77	0.1223
sod-1 A4V^M	UV-k*Ef*-paraquat	123	44.38	
sod-1 A4V^M	H-k*Ef*-paraquat	113	34.78	0.0005

Live *E. faecium* is required for neuroprotection. (A) *sod-1* A4V^M animals were pretreated for 24 hours with live, UV-killed, or heat-killed *E. faecium* before exposure to paraquat-induced oxidative stress. Cholinergic motor neuron integrity was assessed. Data are represented as mean ± SD from three independent experiments. Statistical significance was determined using one-way ANOVA with Tukey's multiple comparison test (***$p < 0.001$). (B) Summary of motor neuron defects from (A) across three independent experiments. The data underlying this Figure can be found in S1 Data.
