# Supplementary material for: A gut-activated NHR-86–CYP pathway mediates the neuroprotective effects of Enterococcus faecium probiotics in a nematode model of amyotrophic lateral sclerosis
Source: PLoS Biol. 2026 Jan 30;24(1):e3003627. doi: 10.1371/journal.pbio.3003627 (PMC12872002; doi:10.1371/journal.pbio.3003627)
Supplement: S4 Fig — (A) Representative images of sod-1 G85RC animals showing intact glutamatergic sensory neurons in the tail labeled with DiD. After paraquat treatment, PHA and PHB neurons fail to take up the dye, indicating degeneration or neuronal loss. Scale bar represents 10 μm. (B) Percentage of defective PHA and PHB neurons in sod-1 G85RC ALS model animals after paraquat treatment. Results from three independent trials are shown. Error bars indicate ±SD. Statistical significance was determined using one-way ANOVA with Tukey’s multiple comparison test (***p < 0.001). (PDF) [file pbio.3003627.s004.pdf]

S4 Fig

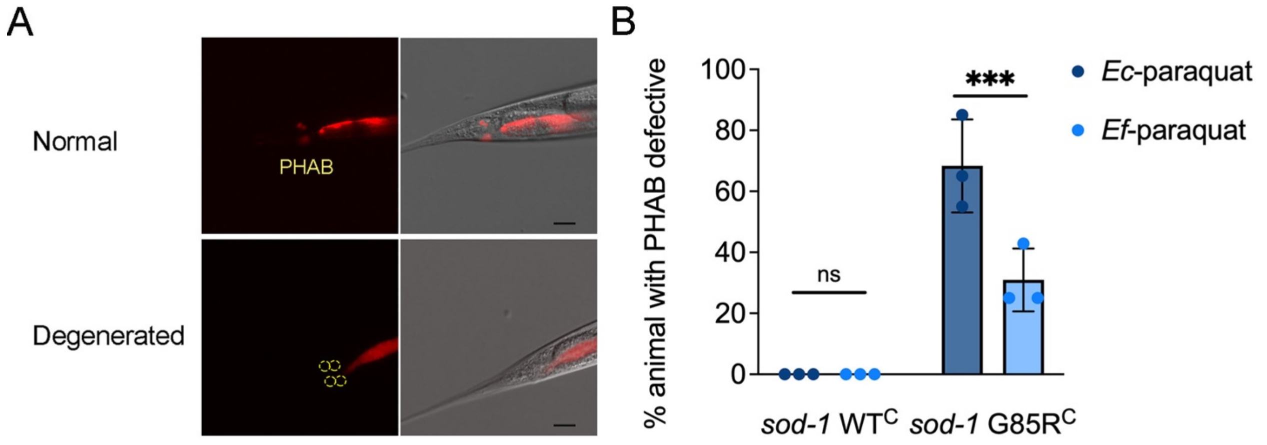

Oxidative stress–induced glutamatergic neuron degeneration in *sod-1* G85R^{C} animals.

(A) Representative images of *sod-1* G85R^{C} animals showing intact glutamatergic sensory neurons in the tail labeled with DiD. After paraquat treatment, PHA and PHB neurons fail to take up the dye, indicating degeneration or neuronal loss. Scale bar represents 10 μm.

(B) Percentage of defective PHA and PHB neurons in *sod-1* G85R^{C} ALS model animals after paraquat treatment. Results from three independent trials are shown. Error bars indicate ±SD. Statistical significance was determined using one-way ANOVA with Tukey's multiple comparison test (***$p < 0.001$). The data underlying this Figure can be found in S1 Data.
