# Supplementary material for: A gut-activated NHR-86–CYP pathway mediates the neuroprotective effects of Enterococcus faecium probiotics in a nematode model of amyotrophic lateral sclerosis
Source: PLoS Biol. 2026 Jan 30;24(1):e3003627. doi: 10.1371/journal.pbio.3003627 (PMC12872002; doi:10.1371/journal.pbio.3003627)
Supplement: S5 Fig — Enrichment analysis of (A) E. faecium upregulated genes and (B) E. faecium downregulated genes using a Wormbase enrichment analysis tool (https://wormbase.org/tools/enrichment/tea/tea.cgi). (PDF) [file pbio.3003627.s005.pdf]

S5 Fig

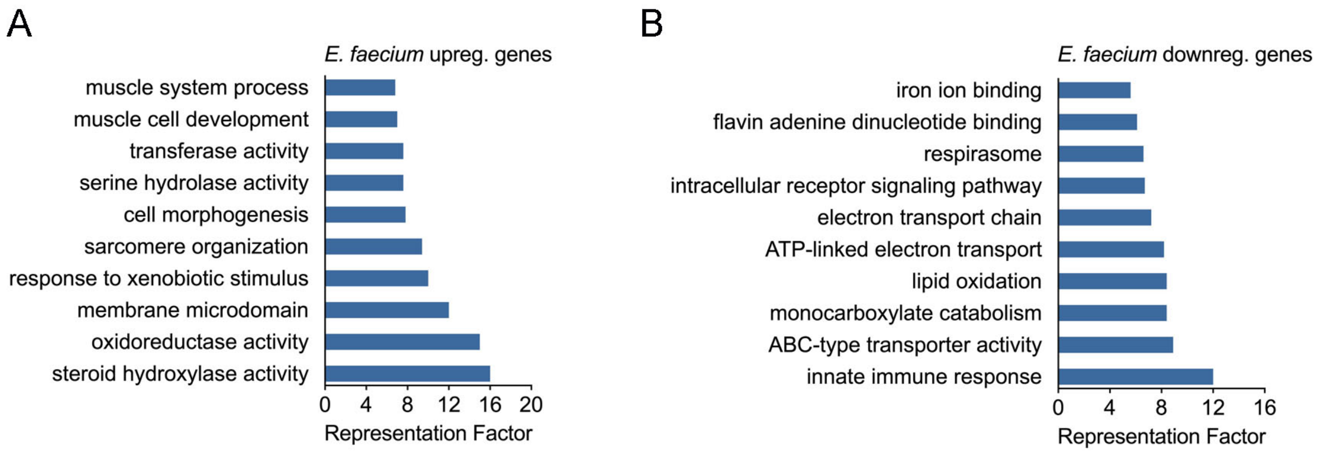

Enrichment analysis of E. faecium regulated genes. Enrichment analysis of (A) *E. faecium* upregulated genes and (B) *E. faecium* downregulated genes using a Wormbase enrichment analysis tool (https://wormbase.org/tools/enrichment/tea/tea.cgi).
