# Supplementary material for: A gut-activated NHR-86–CYP pathway mediates the neuroprotective effects of Enterococcus faecium probiotics in a nematode model of amyotrophic lateral sclerosis
Source: PLoS Biol. 2026 Jan 30;24(1):e3003627. doi: 10.1371/journal.pbio.3003627 (PMC12872002; doi:10.1371/journal.pbio.3003627)
Supplement: S6 Fig — sod-1 A4VM animals were fed control, pmk-1, elt-2, daf-16, or skn-1 RNAi. Motor neuron degeneration was assessed under paraquat-induced oxidative stress, with or without Enterococcus faecium pretreatment. Animals missing at least two neurons were scored as defective. A two-tailed Student t test was performed to compare data between different treatments within the same group. (PDF) [file pbio.3003627.s006.pdf]

S6 Fig

RNAi	Treatment	N	% animals with defective motor neurons	P
Empty vector	*Ec*-paraquat	124	46.77	
Empty vector	*Ef*-paraquat	97	17.53	0.0001
pmk-1	*Ec*-paraquat	106	43.40	
pmk-1	*Ef*-paraquat	97	17.53	0.0006
daf-16	*Ec*-paraquat	103	42.72	
daf-16	*Ef*-paraquat	92	13.04	0.0004
elt-2	*Ec*-paraquat	76	47.37	
elt-2	*Ef*-paraquat	87	19.54	0.0009
skn-1	*Ec*-paraquat	102	46.08	
skn-1	*Ef*-paraquat	102	21.57	0.0005

Motor neuron degeneration under different RNAi knockdowns. *sod-1* A4V^{M} animals were fed control, *pmk-1*, *elt-2*, *daf-16*, or *skn-1* RNAi. Motor neuron degeneration was assessed under paraquat-induced oxidative stress, with or without *E. faecium* pretreatment. Animals missing at least two neurons were scored as defective. A two-tailed Student's t-test was performed to compare data between different treatments within the same group. The data underlying this Figure can be found in S1 Data.
