# Supplementary material for: A gut-activated NHR-86–CYP pathway mediates the neuroprotective effects of Enterococcus faecium probiotics in a nematode model of amyotrophic lateral sclerosis
Source: PLoS Biol. 2026 Jan 30;24(1):e3003627. doi: 10.1371/journal.pbio.3003627 (PMC12872002; doi:10.1371/journal.pbio.3003627)
Supplement: S7 Fig — qRT-PCR validation of cyp genes that were up-regulated with Enterococcus faecium pretreatment (N = 3 biological replicates). Values are expressed as the fold difference compared with sod-1 WTM animals fed on Escherichia coli ± SD by one-way ANOVA with Tukey’s multiple comparison test. (PDF) [file pbio.3003627.s007.pdf]

S7 Fig

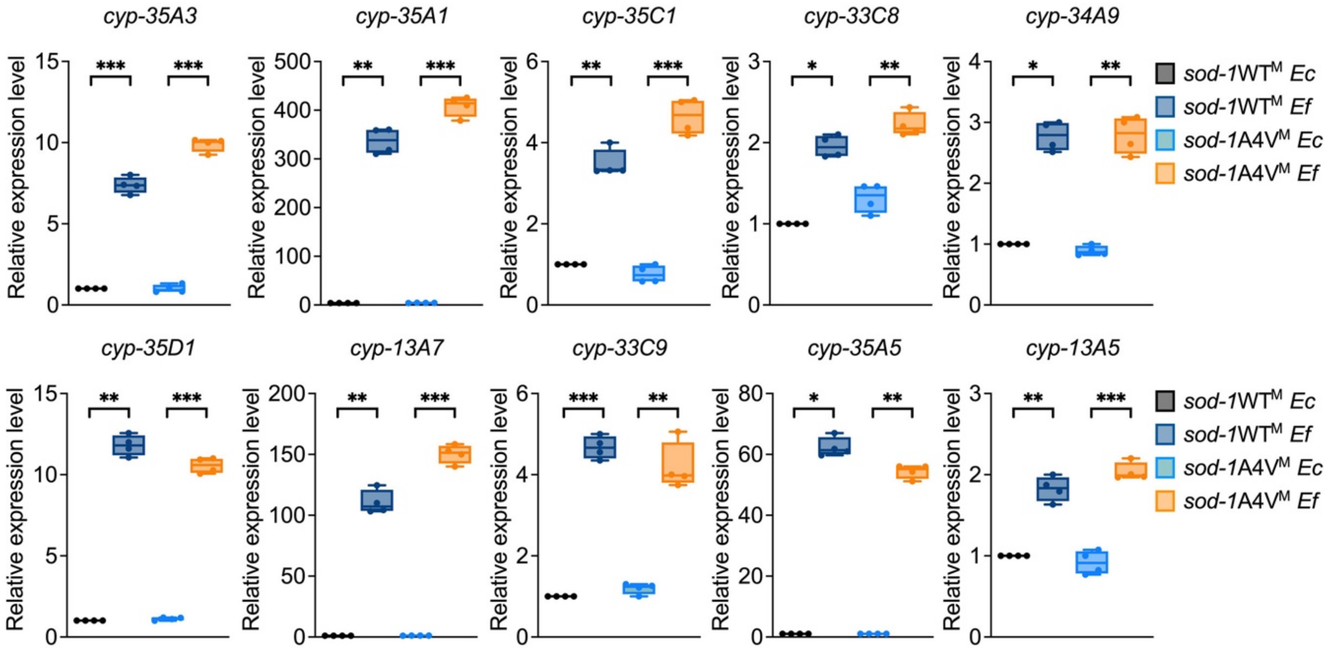

mRNA levels of cytochrome P450 genes. qRT-PCR validation of *cyp* genes that were up-regulated with *E. faecium* pretreatment (N = 3 biological replicates). Values are expressed as the fold difference compared with *sod-1* WT^{M} animals fed on *E. coli* ± SD by one-way ANOVA with Tukey's multiple comparison test. The data underlying this Figure can be found in S1 Data.
