# Supplementary material for: A gut-activated NHR-86–CYP pathway mediates the neuroprotective effects of Enterococcus faecium probiotics in a nematode model of amyotrophic lateral sclerosis
Source: PLoS Biol. 2026 Jan 30;24(1):e3003627. doi: 10.1371/journal.pbio.3003627 (PMC12872002; doi:10.1371/journal.pbio.3003627)
Supplement: S8 Fig — sod-1 A4VM animals were fed control or cyp-35A12345 RNAi. Motor neuron degeneration was assessed under paraquat-induced oxidative stress, with or without Enterococcus faecium pretreatment. Animals missing at least two neurons were scored as defective. One-way ANOVA was performed to compare the data. P values: Empty vector, Ec-paraquat versus Empty vector, Ef-paraquat, P < 0.0001; Empty vector, Ec-paraquat versus cyp-35A12345 RNAi, Ec-paraquat, P = 0.9997; Empty vector, Ef-paraquat versus cyp-35A12345 RNAi, Ef-paraquat, P < 0.0001; cyp-35A12345 RNAi, Ec-paraquat versus cyp-35A12345 RNAi, Ef-paraquat, P = 0.0022. (PDF) [file pbio.3003627.s008.pdf]

S8 Fig

RNAi	Treatment	N	% animals with defective motor neurons
Empty vector	*Ec*-paraquat	180	46.67
Empty vector	*Ef*-paraquat	140	16.43
cyp-35A12345	*Ec*-paraquat	180	47.22
cyp-35A12345	*Ef*-paraquat	216	37.50

Motor neuron degeneration under *cyp-35A12345* RNAi. *sod-1* A4V^{M} animals were fed control or *cyp-35A12345* RNAi. Motor neuron degeneration was assessed under paraquat-induced oxidative stress, with or without *E. faecium* pretreatment. Animals missing at least two neurons were scored as defective. One-way ANOVA was performed to compare the data. P values:

Empty vector, *Ec*-paraquat vs. Empty vector, *Ef*-paraquat, P<0.0001;

Empty vector, *Ec*-paraquat vs. *cyp-35A12345* RNAi, *Ec*-paraquat, P=0.9997;

Empty vector, *Ef*-paraquat vs. *cyp-35A12345* RNAi, *Ef*-paraquat, P<0.0001;

cyp-35A12345 RNAi, Ec-paraquat vs. *cyp-35A12345* RNAi, *Ef*-paraquat, P=0.0022. The data underlying this Figure can be found in S1 Data.
