# Supplementary material for: A gut-activated NHR-86–CYP pathway mediates the neuroprotective effects of Enterococcus faecium probiotics in a nematode model of amyotrophic lateral sclerosis
Source: PLoS Biol. 2026 Jan 30;24(1):e3003627. doi: 10.1371/journal.pbio.3003627 (PMC12872002; doi:10.1371/journal.pbio.3003627)
Supplement: S9 Fig — (A) Screening of nhr genes using (A) cyp-35A3 and (B) cyp-35A5 as reporters. Young adult nhr mutant animals pretreated on E. faecium or Escherichia coli lawns were harvested for RNA extraction and qRT-PCR assay. (N = 3 biological replicates). Values represent fold differences relative to animals fed on E. coli, analyzed by one-way ANOVA with Tukey’s multiple comparisons test, * P < 0.05, ** P < 0.01, *** P < 0.001. (PDF) [file pbio.3003627.s009.pdf]

S9 Fig

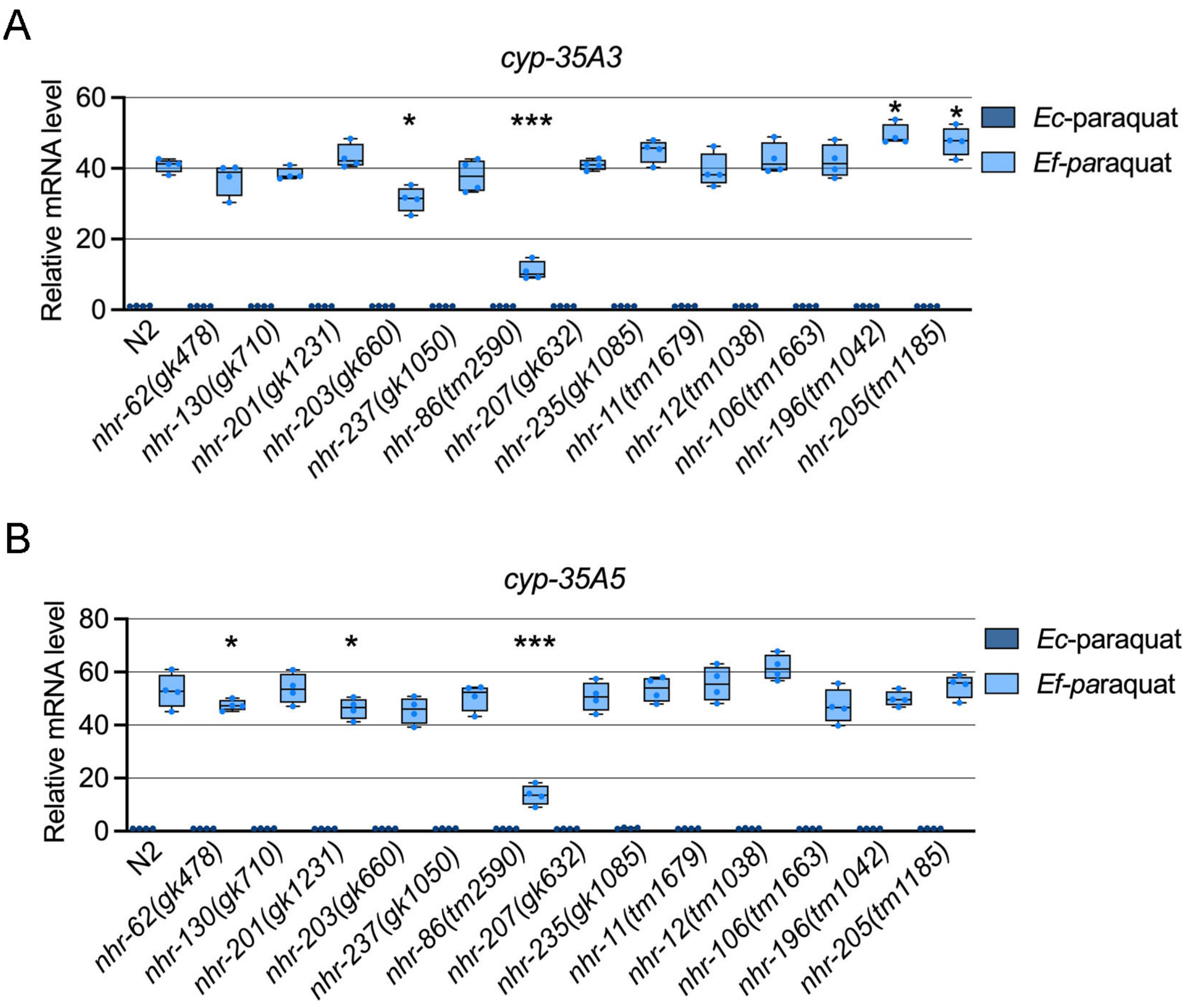

Screen of NHRs required for *cyp* activation by *E. faecium*. (A) Screening of *nhr* genes using (A) *cyp-35A3* and (B) *cyp-35A5* as reporters. Young adult *nhr* mutant animals pretreated on *E. faecium* or *E. coli* lawns were harvested for RNA extraction and qRT-PCR assay. (N = 3 biological replicates). Values represent fold differences relative to animals fed on *E. coli*, analyzed by one-way ANOVA with Tukey's multiple comparisons test, * *P*<0.05, ** *P*<0.01, *** *P*<0.001. The data underlying this Figure can be found in S1 Data.
