# Supplementary material for: A gut-activated NHR-86–CYP pathway mediates the neuroprotective effects of Enterococcus faecium probiotics in a nematode model of amyotrophic lateral sclerosis
Source: PLoS Biol. 2026 Jan 30;24(1):e3003627. doi: 10.1371/journal.pbio.3003627 (PMC12872002; doi:10.1371/journal.pbio.3003627)
Supplement: S10 Fig — sod-1 A4VM animals were fed control, nhr-86 or nhr-203 RNAi. Motor neuron degeneration was assessed under paraquat-induced oxidative stress, with or without Enterococcus faecium pretreatment. Animals missing at least two neurons were scored as defective. One-way ANOVA was performed to compare the mean of nhr-86 RNAi and nhr-203 RNAi with the mean of empty vector pretreated with E. faecium. (PDF) [file pbio.3003627.s010.pdf]

S10 Fig

RNAi	Treatment	N	% animals with defective motor neurons	P
Empty vector	*Ec*-paraquat	109	45.87	
Empty vector	*Ef*-paraquat	177	19.77	0.0001
nhr-86	*Ec*-paraquat	160	44.38	
nhr-86	*Ef*-paraquat	184	34.78	0.0080
nhr-203	*Ec*-paraquat	123	45.53	
nhr-203	*Ef*-paraquat	191	19.37	0.9587

Motor neuron degeneration under *nhr-86* or *nhr-203* RNAi. *sod-1* $A4V^{M}$ animals were fed control, *nhr-86* or *nhr-203* RNAi. Motor neuron degeneration was assessed under paraquat-induced oxidative stress, with or without *E. faecium* pretreatment. Animals missing at least two neurons were scored as defective. One-way ANOVA was performed to compare the mean of *nhr-86* RNAi and *nhr-203* RNAi with the mean of empty vector pretreated with *E. faecium*. The data underlying this Figure can be found in S1 Data.
