# Supplementary material for: A gut-activated NHR-86–CYP pathway mediates the neuroprotective effects of Enterococcus faecium probiotics in a nematode model of amyotrophic lateral sclerosis
Source: PLoS Biol. 2026 Jan 30;24(1):e3003627. doi: 10.1371/journal.pbio.3003627 (PMC12872002; doi:10.1371/journal.pbio.3003627)
Supplement: S11 Fig — Intracellular ROS analysis of sod-1 A4VM animals after cyp-35A12345 knockdown, following Enterococcus faecium pretreatment and paraquat exposure. n = 25. The “n” represents the number of animals in each experiment. N= 3 biological replicates. (PDF) [file pbio.3003627.s011.pdf]

S11 Fig

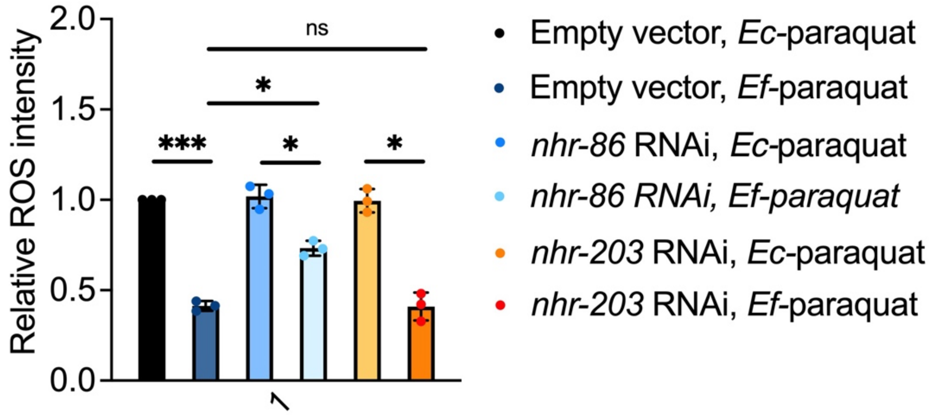

Quantification of DCF fluorescence intensity. Intracellular ROS analysis of *sod-1* A4V^{M} animals after *cyp-35A12345* knockdown, following *E. faecium* pretreatment and paraquat exposure. n=25. The “n” represents the number of animals in each experiment. N = 3 biological replicates. The data underlying this Figure can be found in S1 Data.
