# Supplementary material for: A gut-activated NHR-86–CYP pathway mediates the neuroprotective effects of Enterococcus faecium probiotics in a nematode model of amyotrophic lateral sclerosis
Source: PLoS Biol. 2026 Jan 30;24(1):e3003627. doi: 10.1371/journal.pbio.3003627 (PMC12872002; doi:10.1371/journal.pbio.3003627)
Supplement: S12 Fig — (A) Dot-plot representation of green fluorescence intensity versus TOF of empty vector and nhr-203 RNAi animals exposed to Enterococcus faecium or Escherichia coli followed by paraquat exposure (n = 300). (E) Frequency distribution of green fluorescence in vector or nhr-203 RNAi animals exposed to E. faecium or E. coli followed by paraquat exposure. Three independent experiments were conducted. Empty vector, Ec-paraquat versus Empty vector, Ef-paraquat, P = NS; nhr-203 RNAi, Ec-paraquat versus nhr-203 RNAi, Ef-paraquat, P = NS. (PDF) [file pbio.3003627.s012.pdf]

S12 Fig

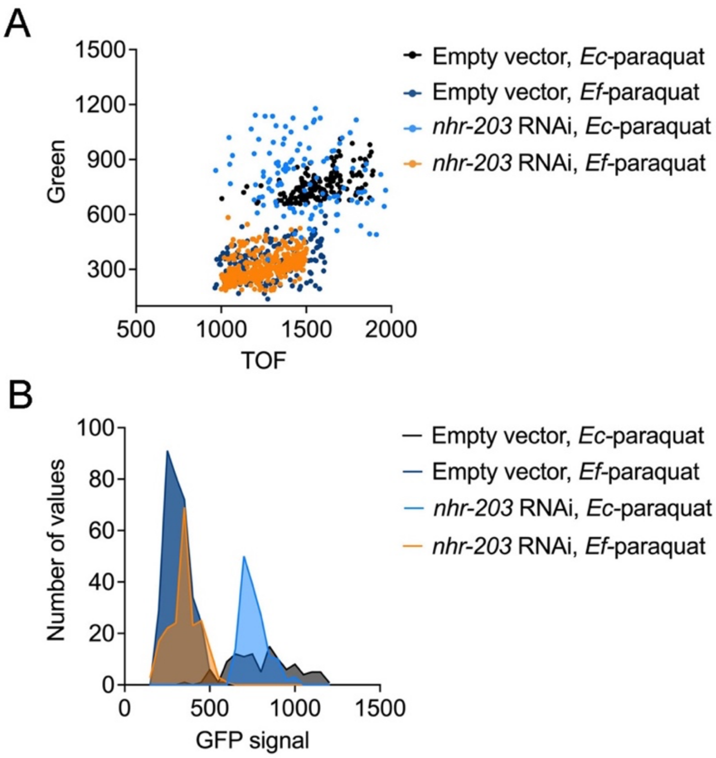

Intracellular ROS analysis of *sod-1* A4V^{M} animals. (A) Dot-plot representation of green fluorescence intensity versus TOF of empty vector and *nhr-203* RNAi animals exposed to *E*. *faecium* or *E*. *coli* followed by paraquat exposure (n = 300). (E) Frequency distribution of green fluorescence in vector or *nhr-203* RNAi animals exposed to *E*. *faecium* or *E*. *coli* followed by paraquat exposure. Three independent experiments were conducted. Empty vector*, Ec*-paraquat versus Empty vector*, Ef*-paraquat, *P* = NS; *nhr-203* RNAi*, Ec*-paraquat versus *nhr-203* RNAi*, Ef*-paraquat, *P* = NS. The data underlying this Figure can be found in S1 Data.
