# Supplementary material for: A gut-activated NHR-86–CYP pathway mediates the neuroprotective effects of Enterococcus faecium probiotics in a nematode model of amyotrophic lateral sclerosis
Source: PLoS Biol. 2026 Jan 30;24(1):e3003627. doi: 10.1371/journal.pbio.3003627 (PMC12872002; doi:10.1371/journal.pbio.3003627)
Supplement: S14 Fig — mRNA level of (A) cyp-35A3 and (B) cyp-35A5 in sod-1 A4VM, sod-1 A4VM;nhr-86(tm2590), and sod-1 A4VM;nhr-86(tm2590);nhr-86p::nhr-86 animals. (N = 3 biological replicates). Values are expressed as the fold difference compared with sod-1 A4VM animals treated by Escherichia coli ± SD by one-way ANOVA with Tukey’s multiple comparisons test, * P < 0.05, ** P < 0.01, *** P < 0.001. (PDF) [file pbio.3003627.s014.pdf]

S14 Fig

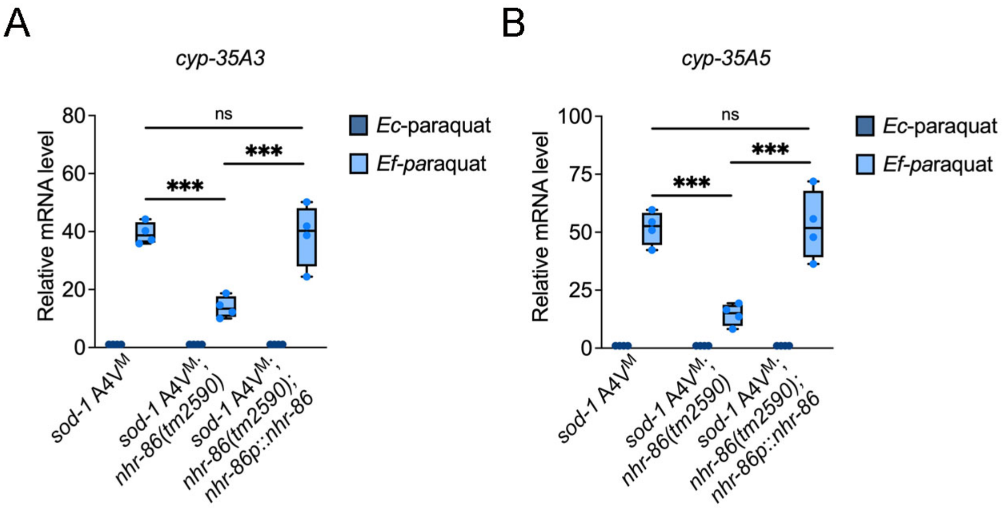

mRNA levels of *cyp* genes with *nhr-86* rescue. mRNA level of (A) *cyp-35A3* and (B) *cyp-35A5* in *sod-1* A4V^{M}, *sod-1* A4V^{M};*nhr-86(tm2590)*, and *sod-1* A4V^{M};*nhr-86(tm2590);nhr-86p*::*nhr-86* animals. (N = 3 biological replicates). Values are expressed as the fold difference compared with *sod-1* A4V^{M} animals treated by *E. coli* ± SD by one-way ANOVA with Tukey's multiple comparisons test, * $P<0.05$, ** $P<0.01$, *** $P<0.001$. The data underlying this Figure can be found in S1 Data.
