# Supplementary material for: A gut-activated NHR-86–CYP pathway mediates the neuroprotective effects of Enterococcus faecium probiotics in a nematode model of amyotrophic lateral sclerosis
Source: PLoS Biol. 2026 Jan 30;24(1):e3003627. doi: 10.1371/journal.pbio.3003627 (PMC12872002; doi:10.1371/journal.pbio.3003627)
Supplement: S15 Fig — (A) Oxidative stress induced cholinergic motor neuron degeneration in sod-1 A4VM animals. sod-1 A4VM;sid-1(qt9); rgef-1p::GFP;rgef-1p::sid-1 animals (AA258) were fed control, nhr-86, dnc-1, or smn-1 RNAi. After growing to young adults on RNAi plates, the animals were treated with Enterococcus faecium or Escherichia coli for 24 hours before a 24-hour paraquat exposure. Cholinergic motor neuron integrity was accessed by scoring for the absence of at least two cholinergic motor neurons posterior to the vulva. * P < 0.05, ** P < 0.01, *** P < 0.001, two-way ANOVA. (B) Summary of motor neuron defects from (A) across three independent experiments. (PDF) [file pbio.3003627.s015.pdf]

S15 Fig

A

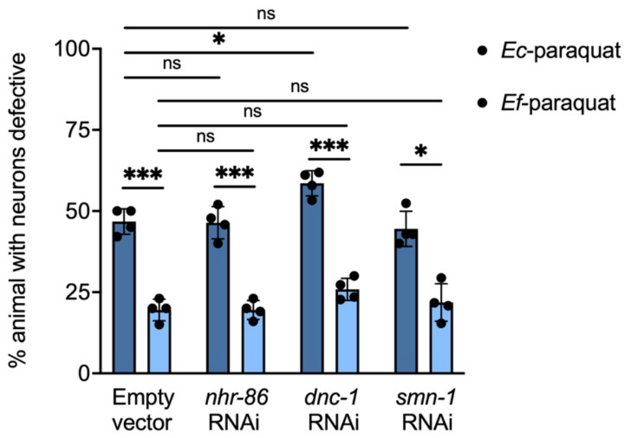

B

RNAi	Treatment	N	% animals with defective motor neurons	P
Empty vector	*Ec*-paraquat	75	46.67	
Empty vector	*Ef*-paraquat	73	19.18	0.0001
nhr-86	*Ec*-paraquat	90	44.44	
nhr-86	*Ef*-paraquat	87	32.18	0.0047
dnc-1	*Ec*-paraquat	85	58.90	
dnc-1	*Ef*-paraquat	95	25.00	0.0024
smn-1	*Ec*-paraquat	90	44.44	
smn-1	*Ef*-paraquat	90	21.11	0.0205

Motor neuron degeneration under different RNAi knockdowns. (A) Oxidative stress induced cholinergic motor neuron degeneration in *sod-1* A4V^{M} animals. *sod-1* A4V^{M};*sid-1(qt9)*; *rgef-1p*::GFP;*rgef-1p*::*sid-1* animals (AA258) were fed control, *nhr-86*, *dnc-1*, or *smn-1* RNAi. After growing to young adults on RNAi plates, the animals were treated with *E. faecium* or *E. coli* for 24 hours before a 24-hour paraquat exposure. Cholinergic motor neuron integrity was accessed by scoring for the absence of at least two cholinergic motor neurons posterior to the vulva. * *P*<0.05, ** *P*<0.01, *** *P*<0.001, 2way ANOVA. (B) Summary of motor neuron defects from (A) across three independent experiments. The data underlying this Figure can be found in S1 Data.
