# Supplementary material for: A gut-activated NHR-86–CYP pathway mediates the neuroprotective effects of Enterococcus faecium probiotics in a nematode model of amyotrophic lateral sclerosis
Source: PLoS Biol. 2026 Jan 30;24(1):e3003627. doi: 10.1371/journal.pbio.3003627 (PMC12872002; doi:10.1371/journal.pbio.3003627)
Supplement: Attachment — Submitted filename: Point by point response.docx [file pbio.3003627.s025.docx]

REVIEWERS' COMMENTS:

Reviewer #1:

In this study, Sang et al., found that the probiotic *E. faecium* appears to be protect motor neuron degeneration through NHR-86, which activates the expression of cytochrome P450, which reduces intracellular levels of ROS to reduce oxidative stress and confer neuroprotection. Although the study is interesting in identifying NHR-86 as a key factor, it falls short in elucidating the mechanisms underlying the activation of NHR-86. Below are some comments that can hopefully help improve the manuscript.

Response: We thank Reviewer #1 for the thoughtful and constructive critique of our manuscript. The reviewer highlights an important gap in our original submission regarding the mechanism by which *E. faecium* activates NHR-86. In the revised manuscript, we have added new experimental data and analyses that directly address these concerns. These additions include testing whether *E. faecium* regulates *nhr-86* expression, assessing the intestinal site of action of NHR-86 through tissue-specific rescue and RNAi approaches, evaluating whether NHR-86 overexpression alone is sufficient for protection, and integrating ChIP-seq data to clarify the relationship between NHR-86 target genes and the *E. faecium* transcriptional response. Collectively, these results provide a clearer framework for understanding how NHR-86 functions downstream of *E. faecium* and strengthen the mechanistic basis of the study.

Major

1.	Is the expression of *nhr-86* regulated by *E. faecium*? This can be easily tested by treating the *nhr-86*::GFP strain with *E. faecium*.

Response: Our new qRT-PCR data (New Fig S13) show that *E. faecium* does not alter *nhr-86* expression. This is consistent with our RNA-seq. This information has been included in the revised manuscript: Lines 249-251

2.	In what tissue does *nhr-86* function to confer neuroprotection? The authors mentioned that *nhr-86* is expressed in hypodermis, intestine, and head neurons. So, the authors can use tissue-specific promoter to drive *nhr-86* expression and identify the site of action for *nhr-86*. One likely place is the intestine, since it appears to be where *nhr-86* is expressed the strongest. If that is the case, the author may want to explain or hypothesize how intestinal activation of NHR-86 and CYP genes confer protection on motor neurons through cell-non-autonomous mechanisms.

Response: We sincerely appreciate the reviewer’s suggestions. To experimentally address this issue rather than hypothesize on the mechanism, we planned both tissue-specific RNAi and tissue-specific rescue experiments to determine in which tissue NHR-86 functions to confer neuroprotection. However, we encountered significant technical challenges during these attempts.

Challenges in crossing tissue-specific RNAi strains:

Crossing tissue-specific RNAi strains into the *sod-1* A4V^M^ ALS model was hindered by slow growth, reduced brood size, and partial embryonic lethality, resulting in very low frequencies of the desired genotypes. Neuron-specific RNAi strains were obtained, but gut-specific strains could not be generated.

Rescue experiments and technical limitations:

Rescue experiments were challenging because deleting *nhr-86* in *sod-1* A4V^M^ required extensive crossing, and initial RFP-based rescue constructs produced transgenic animals that did not survive beyond the first generation.

Finally, we successfully achieved neuronal-specific RNAi and rescue using a gut-specific promoter in the *sod-1* A4V^M^ animals. Neuron-specific knockdown of *nhr-86* in *sod-1* A4V^M^ animals had no effect on *E. faecium*–mediated neuroprotection (new Fig. S15), whereas intestine-specific expression of NHR-86 under the *vha-6* promoter fully restored protection against motor neuron degeneration, rescued *cyp-35A1/3/5* expression, and restored survival and locomotion (new Fig 7A–7E, new Fig. S16). These results suggest that NHR-86 primarily functions in the intestine to mediate neuroprotection. Lines 273-298

We added the following paragraph to the Discussion: “In our model, the intestinal probiotic *E. faecium* protects motor neurons during oxidative stress induced by paraquat. *E. faecium* induces the expression of CYP enzymes, and the intestinal function of the nuclear hormone receptor NHR-86 is required for CYP induction and neuroprotection. These CYPs reduce intracellular ROS generated by paraquat-induced oxidative stress. Because ROS are diffusible, lowering their ROS levels in the intestine may also reduce oxidative stress in neighboring motor neurons. Thus, the probiotic may indirectly protect neurons by enhancing intestinal antioxidant capacity. These findings provide direct experimental evidence that intestinal response to microbial signals can modulate neuronal resilience under environmental oxidative stress.” Lines 336-345

3.	One the other hand, does overexpression of *nhr-86* itself confers protection against motor neuron degeneration under *E. coli* diet? This is a crucial experiment, because it can test whether the expression level of *nhr-86* itself is sufficient for neuroprotection or it must be activated by some ligands present in the probiotic *E. faecium*.

Response: Overexpression of NHR-86 alone did not confer neuroprotection or induce *cyp* expression on an *E. coli* diet (new Fig 6B and 6C), indicating that NHR-86 activation depends on specific signals provided by *E. faecium*. Lines 269-272

4.	Is there any transcriptomic data on *nhr-86* mutants? If available, the authors can compare the NHR-86-regulated genes with *E. faecium*-regulated genes to see if NHR-86 is indeed the major effector downstream of *E. faecium*. A simple search of the literature led to this paper (PMID: 30668573). It seems Peterson et al., has even done ChIP-Seq on NHR-86. These data gave the possibility for the above analysis. The authors can also look at the CYP genes in the ChIP-seq data to see if they are bound by NHR-86.

Response: We thank the reviewer for this suggestion. We analyzed the published NHR-86 ChIP-seq dataset to assess whether NHR-86 is a major effector downstream of *E. faecium*. Our analysis revealed a notable overlap between *E. faecium*–induced genes and NHR-86–bound targets, including CYP genes such as *cyp-35A1* and *cyp-35A5*, as well as multiple other genes (updated Table S1). These results further support that NHR-86 contributes directly to the transcriptional program activated by *E. faecium*. Lines 252-258

5.	Can *E. faecium* colonize the gut of *C. elegans*? This can be tested through smFISH staining like previously performed (PMID: 32669368). Can UV-killed *E. faecium* still confer protection. It will be important to know whether *E. faecium* needs to be alive and colonize the gut of *C. elegans* to confer protection or it can simply be served as a diet to confer protection.

Response: We thank the reviewer for this important point. Prior work has demonstrated that live *E. faecium* can stably colonize the *C. elegans* intestine (PMID: 29436902). Consistent with this requirement for viability, we found that both UV-killed and heat-killed *E. faecium* failed to provide neuroprotection (new Fig S2A and S2B). These results indicate that *E. faecium* viability and gut colonization are necessary for its protective effect, rather than merely serving as a dietary supplement. Lines 129-131

6.	In terms of the disease modeling, does SOD-1 A4V mutants show motor neuron loss or defects without paraquat treatment?

Response: The SOD-1 A4V^M^ single-copy model does not exhibit cholinergic motor neuron loss or functional defects under basal conditions in young adult animals, although it can form protein inclusions in motor neurons (PMID: 30296255). Lines 111-114

7.	From Figure 1C, it seems that if SOD-1 is WT, *E. faecium* does not confer protection. So, the SOD1 A4V mutants is required to see the effects of *E. faecium*. Since the authors are trying to model ALS using the mutant, they must at least discuss how the SOD mutation contributes to the hypersensitivity to oxidative stress and how it interacts with *E. faecium*. For example, is the *E. faecium*-induced CYP upregulation not sufficient to counter the oxidative stress in the SOD WT condition? Or is it because the phenotype is not severe enough to see any difference?

Response: *E. faecium*-mediated neuroprotection is only apparent in the context of ALS-associated SOD-1 mutations, which sensitize motor neurons to oxidative stress. This genotype-dependence is discussed in the revised Discussion section. Lines 346-351: “*E. faecium*-mediated neuroprotection is observed only in mutants with heightened stress sensitivity, such as *sod-1* A4V^M^, but not in *sod-1* WT^M^ animals, where baseline stress is insufficient to induce degeneration. These findings indicate that ALS-linked SOD-1 mutations prime neurons for stress-induced degeneration, providing a context in which probiotics can exert measurable protective effects and highlighting the relevance of the *sod-1* A4V^M^ model for studying ALS-related neurodegeneration.”

8.	"Since the innate immune response cluster is among the most down-regulated, we speculated that knocking down immune pathways would decrease *E. faecium*-mediated protection against motor neuron degeneration." The logic of this sentence is flawed. If innate immune genes are downregulated by *E. faecium*, that most likely means the innate immune genes are neurotoxic, so downregulating them can facilitate neuroprotection. So, the prediction is that overexpression of these innate immune gene would block *E. faecium*-mediated neuroprotection. So, the experiments in Figure 3A does not mean much. I will suggest overexpressing these genes to test their effects.

Response: We apologized for the typo. The sentence should have read “Since the innate immune response cluster is among the most down-regulated, we speculated that knocking down immune pathways may protect against motor neuron degeneration.” We corrected this section in the revised manuscript. The lack of protection by inhibition of immune pathways in the absence of *E. faecium* suggests that they are not part of the mechanism. Our current data indicate that CYP enzymes are the essential effectors mitigating ROS and preserving motor neuron integrity. Lines 180-192

Minor

1.	What promoter was used to drive the expression of *sod-1* WT and A4V? This is not very clear to me. Is it expressed in all motor neurons?

Response: *unc-17* drives expression specifically in cholinergic motor neurons*.* Lines 121-123

2.	Figure 1A, I suggest adding some labels on the images to show which one is *Ec*-treated and which ones are *Ef*-treated.

Response: Both images in Fig 1A depict animals treated with *E. coli*. This information has been clarified in the Figure Legend. Lines 551-553

3.	When examining neuronal loss, did the author notice whether the number of neurons that are defective are different between *Ec* and *Ef*-treated animals (when they are classified as animals with neurons defective)?

Response: Animals were classified as “defective” when more than two motor neurons were lost. In this study, we did not further subdivide defective animals based on the number of neurons missing. Our analysis focused on the proportion of animals that crossed the threshold for degeneration, whick clearly differed between *E. coli* and *E. faecium*. Although we did not quantify the extent of neuronal loss within defective animals, the reduction in the fraction of defective animals under *E. faecium* indicates a protective effect.

4.	For Figure 5D and E, there need to be some statistical analysis on the results.

Response: Statistical analysis is provided in the Figure Legend. Fig 5D (dot plot) and 5E (frequency distribution) present the same data in different formats to highlight complementary aspects of the results. Line 636-640

5.	"We performed an RNAi screen targeting 13 nhr genes responsible for regulating cyp genes" Judging from Figure 5A, this is not a RNAi screen but rather a candidate mutant screen.

Response: Fig 5A used candidate NHR mutants, not RNAi; the text has been corrected. Lines 231-234

Reviewer #2:

Summary

This manuscript reports that exposure to *E. faecium* suppresses oxidative damage in a *C. elegans* SOD1 ALS model with results from RNA sequencing and other studies that implicate nuclear hormone receptor NHR-86 regulation of *cyp* gene expression in the intestine as a key player in this process. The studies are undertaken with appropriate controls, the conclusions are reasonable, and the manuscript is of high-quality with admirably clear descriptions. There are a few points that are missing in the text (noted below).

The manuscript would have much more impact in the ALS or gut/brain axis field if the authors included studies that 1) address the generality of *E. faecium* suppression in other ALS or SOD1 ALS models and/or 2) determine if catecholaminergic signaling is implicated in communication between the intestine and the gut. Without these, the impact of the paper is somewhat diminished, but still of interest to researchers interested in oxidative stress response.

Response: We thank the reviewer for the thoughtful summary and for highlighting important directions to enhance the impact of this work. We agree that evaluating the generality of *E. faecium*’s neuroprotective effects and exploring how intestinal oxidative responses are functionally connected to motor neuron degeneration are valuable goals. In this revision, we have added new data showing protection of glutamatergic neurons and expanded the Discussion to address the mechanistic link between intestinal oxidative responses and neuronal resilience. We believe these additions strengthen the manuscript and provide a clear framework for subsequent studies.

Major comments

1. Evidence that exposure to *E. faecium* suppresses sod-1A4V motor neuron defects is convincing. But in ALS patients, including SOD1A4V patients, glutamatergic neurons are also lost. Does the sod-1A4V model also show glutamatergic neuron loss and is this suppressed by *E. faecium*? Is there another model that allows assessment of glutamatergic neurons? Probing this would considerably increase the impact of the manuscript.

Response: We performed additional experiments to address this important point. Specifically, we tested whether *E. faecium* protects glutamatergic neurons using a *sod-1* G85R^M^ ALS model, which recapitulates glutamatergic neuron degeneration under oxidative stress (PMID: 33474528). We assessed sensory neurons PHA and PHB and found that *E. faecium* treatment significantly reduced degeneration in these neurons. These results demonstrate that the neuroprotective effects of *E. faecium* extend beyond cholinergic motor neurons and highlight its broader potential to enhance neuronal resilience under stress.

The revised manuscript (Line 140-146) reads: “Because ALS patients carrying the SOD1 A4V mutation also exhibit glutamatergic neuron loss, we examined whether *E. faecium* protects these neurons. Using a *sod-1* G85R^M^ animal model, which recapitulates glutamatergic neuron degeneration under oxidative stress (PMID: 33474528), we assessed sensory neurons PHA and PHB. *E. faecium* treatment reduced degeneration in these neurons (new Fig S4A and S4B), showing that its neuroprotective effects extend beyond cholinergic motor neurons. These results highlight the broader potential of the probiotic to enhance neuronal resilience under stress.”

2. The studies shown in Figures 3 and 5 used RNAi by feeding to determine if knockdown of transcription factors decreased *E. faecium* neuroprotection. Most readers will not know that C. elegans neurons are insensitive to RNAi by feeding and that conclusions drawn about these genes from these studies only applies to a possible role for these genes in the intestine and other non-neuronal tissues. This caveat should be explicitly mentioned in both the main text and in the figure legend.

Response: We thank the reviewer for this important point. We agree that RNAi by feeding is largely ineffective in most *C. elegans* neurons and therefore primarily targets intestinal and other non-neuronal tissues. Consequently, our conclusions from Fig 3, 4, and 5 are restricted to the non-neuronal roles of these genes in mediating *E. faecium*–dependent neuroprotection. We have now explicitly added clarifying statements to both the main text and Methods. Lines 186-188, Lines 459-461

3. How oxidative response in the intestine is functionally connected to neurodegeneration of motor neurons is not experimentally addressed and probing this would considerably increase the impact of the manuscript. The manuscript discussion includes a discussion paragraph suggesting two possible mechanisms, citing general reviews in the field of gut/brain axis: (1) microbial-derived short-chain fatty acids and (2) catecholamine neurotransmission changes. The latter should be straightforward to test experimentally using *C. elegans* mutant strains or RNAi, especially as the signal may be arising from the intestine.

Response: We appreciate the reviewer’s insightful comment. While we agree that probing the mechanistic link between intestinal oxidative responses and motor neuron degeneration would further strengthen the manuscript, our current study provides direct evidence of such a connection through intestinal CYP induction and NHR-86 function. These findings establish a clear pathway by which intestinal antioxidant capacity influences neuronal resilience under oxidative stress. We have added the following paragraph to the Discussion (Lines 336-345): “In our model, the intestinal probiotic *E. faecium* protects motor neurons during oxidative stress induced by paraquat. *E. faecium* induces the expression of CYP enzymes, and the intestinal function of the nuclear hormone receptor NHR-86 is required for CYP induction and neuroprotection. These CYPs reduce intracellular ROS generated by paraquat-induced oxidative stress. Because ROS are diffusible, lowering their ROS levels in the intestine may also reduce oxidative stress in neighboring motor neurons. Thus, the probiotic may indirectly protect neurons by enhancing intestinal antioxidant capacity. These findings provide direct experimental evidence that intestinal response to microbial signals can modulate neuronal resilience under environmental oxidative stress.”

Minor Comments

Does exposure to *E. faecium* protect in nonSOD1 ALS or FTD models? It's not critical to include this, but readers will ask this obvious question.

Response: We thank the reviewer for this question. While our primary focus was on the SOD1 A4V model, we also tested *E. faecium* in the *sod-1* G85R^M^ ALS model, which recapitulates glutamatergic neuron degeneration under oxidative stress. *E. faecium* reduced degeneration in these neurons (new Fig S4B), demonstrating that its neuroprotective effects extend beyond cholinergic motor neurons and a single SOD1 mutation. We have not yet evaluated non-SOD1 ALS models or frontotemporal dementia (FTD) models, but we recognize this as an important future direction. A statement highlighting the need to assess probiotic protection across diverse neurodegeneration models has been added to the Discussion. Lines 351-356

The Introduction states that "Over forty gene mutations have been linked to ALS, with the most prevalent occurring in the sod-1 gene…", but the fraction of ALS cases caused SOD1 mutations is very low compared to other genetic causes of ALS. Correction and citation adding another citation would be helpful.

Response: Thank you for the comment. We have revised the Introduction to clarify the actual prevalence of SOD1 mutations in ALS and added appropriate citations to support this correction. Lines 38-42

If *C. elegans* genes/pathways have been previously implicated in intestinal protection of C. elegans neurons, then those should also be cited/discussed in the discussion section.

Response: We appreciate this comment. This point has been addressed in the revised Discussion, where we now cite and discuss *C. elegans* intestinal pathways previously implicated in neuronal protection. Lines 314-327

The Methods section states that "All samples were run in triplicate". Were three independent biological replicates used? Please clarify for the reader.

Response: The Methods section has been revised to clarify that results are based on three independent experiments, each performed in triplicate. Line 493

Were researchers blinded as to genotype/treatment when capturing/selecting images for analysis or for any scoring that was undertaken manually? An explicit statement in the Methods section is needed.

Response: The Methods section has been revised to explicitly state that researchers were blinded to genotype and treatment during image acquisition and manual scoring. Lines 422-424
